# Is early bilateral compression ultrasonography and D-dimer monitoring appropriately for prophylaxis and diagnosis of deep venous thrombosis after cesarean section women: a single-center observation study of Chinese Han population

**DOI:** 10.1186/s12884-024-06372-8

**Published:** 2024-03-07

**Authors:** Xiuying Chen, Haiyan Jiang, Aiping Zhou, Quan Zhang, Minmin Du, Yun Sun, Baihui Zhao

**Affiliations:** 1 Department of Obstetrics and Gynecology, The Fourth Affiliated Hospital, Zhejiang University School of Medicine, NO. 1 Shangcheng Road, Yiwu, Zhejiang 322300 China; 2 Department of Obstetrics and Gynecology, The Affiliated Taian City Central Hospital of Qingdao University, 29 Longtan Road, Taian, Shandong 271000 China

**Keywords:** Bilateral compression ultrasonography, D-dimer, Prophylaxis, Diagnosis, Deep venous thrombosis, Cesarean section

## Abstract

**Background:**

Venous thromboembolism (VTE) is most prevalent among parturients following a cesarean section (CS). The objective of this study was to assess the practical utility of bilateral compression ultrasonography (CUS) of the lower limbs, coupled with D-dimer monitoring, in the early diagnosis of VTE within the Han Chinese population.

**Methods:**

Our prospective observational study included 742 women who underwent CUS and D-dimer testing on the first day post-CS. Subsequently, telephone or outpatient follow-ups were conducted until 42 days postpartum. States of hypercoagulation and thrombosis, as indicated by CUS, were classified as CUS abnormal. A D-dimer level ≥ 3 mg/l was considered the D-dimer warning value. Early ambulation and mechanical prophylaxis were universally recommended for all parturients post-CS. A sequential diagnostic strategy, based on the 2015 RCOG VTE risk-assessment tool, was employed. Therapeutic doses of low-molecular-weight heparin (LMWH) were administered for the treatment of thromboembolic disease. Prophylactic doses of LMWH were given for VTE prophylaxis in parturients with hypercoagulative status accompanied by D-dimer levels ≥ 3 mg/l. All high-risk women (RCOG score ≥ 4 points) were additionally treated with preventive LMWH. Statistical analyses were conducted using the R statistical software, with a two-sided P value < 0.05 considered statistically significant.

**Results:**

Fifteen cases of VTE and 727 instances without VTE were observed. The overall VTE rate post-CS was 2.02% (15/742), with 66.7% (10/15) being asymptomatic. Eleven patients received a VTE diagnosis on the first postpartum day. Among the 41 parturients exhibiting hypercoagulation ultrasound findings and D-dimer levels ≥ 3 mg/l, despite receiving pharmacological VTE prophylaxis with LMWH, 4.88% (2/41) in the high-risk group were eventually diagnosed with VTE. A total of 30.86% (229/742) exhibited normal ultrasound findings and D-dimer levels < 3 mg/l on the first day post-CS, with no VTE occurrences in the postpartum follow-up. According to RCOG’s recommendation, 78.03% (579/742) of cesarean delivery women should receive prophylactic anticoagulation, while only 20.62% (153/742) met our criterion for prophylactic anticoagulation.

**Conclusion:**

The strategy of timely routine bilateral CUS and D-dimer monitoring is conducive to the early diagnosis and treatment of VTE, significantly reducing the use of LMWH in the Chinese Han population.

## Background

Venous thromboembolism (VTE) stands as the third most prevalent cause of mortality globally, constituting approximately 12% of deaths, according to the World Health Organization [1]. The umbrella term VTE encompasses both deep vein thrombosis (DVT) and pulmonary embolism (PE). Pregnancy and the postpartum period are firmly established as risk factors for VTE [2]. Pregnancy, a distinctive state for women, witnesses substantial alterations in the functioning of key homeostatic systems within a relatively brief timeframe. While inherently physiological, these changes elevate the risk of venous thromboembolism by nearly sixfold [1]. VTE in the female demographic manifests at a youthful age of 40 in 50% of cases, with 20% of these cases linked to pregnancy, as per data from the RIETE [3]. Survey findings suggest that the incidence of pregnancy-related VTE in China, the United Kingdom, and the United States is 0.068%, 0.107%, and 0.066%, respectively [4–6]. Currently, thrombosis and thromboembolism stand as the primary causes of maternal morbidity and mortality [7]. In the U.S., VTE-related deaths contribute to 9–10% of all pregnancy-related mortalities [8], while in the United Kingdom, pulmonary embolism results in the deaths of 5–10 women during pregnancy and the puerperium annually [9].

VTE exhibits a higher prevalence during the postpartum period compared to the antepartum phase [10, 11]. Cesarean section (CS), with an incidence of 2.6 per 1,000 CS, emerges as the predominant risk factor for postnatal thrombosis cases [12–14].

The optimal approach to thromboprophylaxis in pregnant women remains uncertain, with available options encompassing early ambulation, mechanical interventions, and/or pharmacological agents. The efficacy of thromboprophylaxis specifically for cesarean births has not been adequately explored in sufficiently powered, randomized trials that comprehensively evaluate both benefits (prevention of VTE) and potential harms (wound or bleeding complications [15, 16]). Timely and accurate diagnosis, along with effective prevention of VTE, assumes paramount importance [17, 18]. Discrepancies exist among international guidelines regarding the selection of patients for thromboprophylaxis after cesarean section (CS), primarily due to the unclear optimal threshold for initiating pharmacologic thromboprophylaxis and the optimal duration of therapy [7].

The 2015 RCOG Guidelines were employed to assess the risk of postpartum VTE. According to these guidelines, women undergoing CS delivery should receive pharmacologic prophylaxis for 10 days, except for those with an elective cesarean section and no additional risk factors. Notably, a substantial portion of CS patients clinically meets the criteria for pharmacologic VTE prophylaxis but does not receive it [19]. Therefore, the prompt and effective identification of women who would derive the greatest benefit from preventive anticoagulation is crucial. In 2021, the Obstetrics and Gynecology Branch of the Chinese Medical Association recommended the use of intermittent pneumatic compression devices or plantar vein pumps for at least 2 days post-cesarean section to prevent thrombus formation. Additionally, it was advised to conduct compression ultrasonography (CUS) beforehand to rule out the presence of VTE. While D-dimer levels alone are not recommended as the basis for VTE prevention and treatment, monitoring D-dimer levels is deemed necessary during the treatment of patients with a clearly diagnosed VTE.

The objective of this study was to investigate the practical utility of bilateral CUS of the lower limbs (referred to as ultrasound in the text) in conjunction with D-dimer monitoring for the prevention and early diagnosis of venous thromboembolism (VTE) following cesarean section (CS) and to determine the suitability of this strategy within the Han Chinese population.

## Methods

### Study design

This study constituted a prospective single-center observational investigation based on our hospital’s pregnancy registry. Approval for the study was granted by the Institutional Review Board of the Fourth Affiliated Hospital of Zhejiang University (Protocol K2022109). Given the registry nature of the study, patients provided verbal notification instead of written informed consent by the Institutional Review Board of the Fourth Affiliated Hospital of Zhejiang University. The study spanned from January 2020 to March 2022, involving pregnant women admitted to The Fourth Affiliated Hospital of Zhejiang University, as identified in the computer-based clinical management system. Inclusion criteria comprised women who underwent ultrasound examination and D-dimer testing on the first day post-cesarean section. Demographic data, obstetric details, additional surgeries accompanying cesarean section (e.g., sterilization, ovarian cyst removal, uterine packing hemostasis), and the results of D-dimer and ultrasound were meticulously recorded and reviewed. Exclusion criteria encompassed vaginal deliveries, significant risk factors for postpartum VTE (e.g., prior VTE, thrombophilia, or the absence of ultrasound examination within 24 h after cesarean section. Ultrasonic findings beyond normal and thrombosis (e.g., slow blood flow, hypercoagulative status) were classified as abnormal. Diagnosis of deep vein thrombosis (DVT) relied on ultrasound, while pulmonary embolism (PE) diagnosis was based on computed-tomography pulmonary angiography (CTPA). A sequential diagnostic strategy, incorporating the 2015 RCOG VTE risk-assessment tool, routine D-dimer measurement, and ultrasound examination post-cesarean section, was implemented, even in the absence of symptoms. Postpartum VTE risk after cesarean section was stratified into three levels, adhering to the 2015 RCOG Guidelines: high risk (postpartum score ≥ 4 points), intermediate risk (2–3 points), and low risk (0–1 points). D-dimer concentrations (mg/L) were expressed in fibrinogen-equivalent units Following Che et al.‘s study, a D-dimer level ≥ 3 mg/l served as the VTE alarm value in our investigation [20]. Early ambulation and mechanical prophylaxis (graduated elastic compression stockings and intermittent pneumatic compression) were universally recommended post-cesarean section. Additionally, high-risk parturients (RCOG score ≥ 4) received pharmacological VTE prophylaxis with low-molecular-weight heparin (LMWH) in the absence of contraindications. Medium-risk parturients with abnormal ultrasound findings and D-dimer levels ≥ 3 mg/l were also administered pharmacological VTE prophylaxis with LMWH. Detection of DVT prompted routine CTPA checks to explore the presence of PE. Both DVT and PE were managed by vascular specialists.

### Statistical analysis

Continuous variables were articulated as means ± standard deviation, while discrete variables were expressed as percentages. Baseline clinical characteristics underwent statistical comparison through Student’s t or Fisher’s exact tests, as deemed appropriate. Categorical variables were conveyed as numerical values and percentages (%). Analysis of categorical variables employed a chi-squared test. The statistical analysis utilised R (version 4.0.4; R Development Core Team) statistical software. A two-sided P value < 0.05 was considered indicative of statistical significance.

## Results

### Clinical characteristics of the study population

**Fig. 1:**
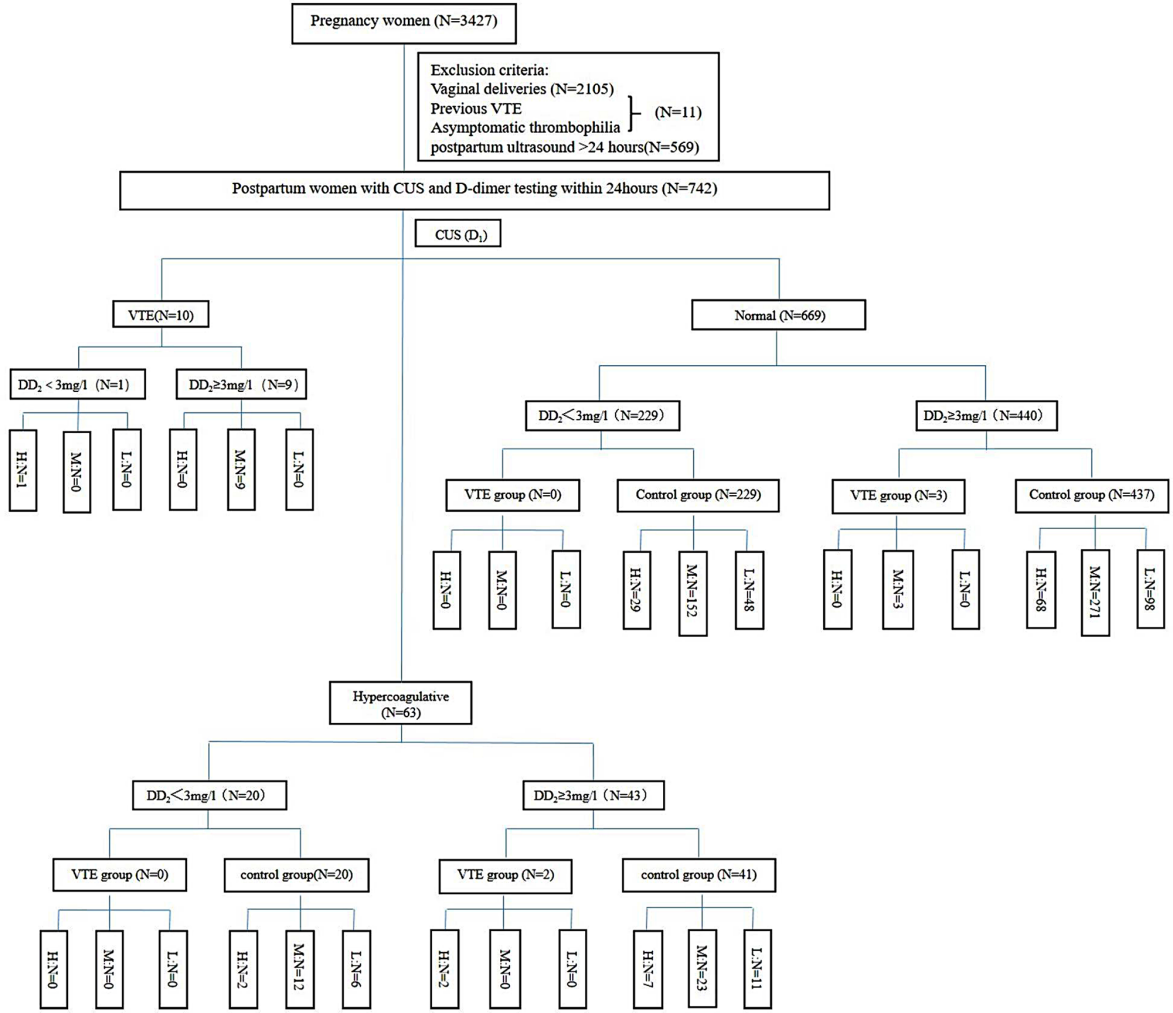
Flow chart. *Note* RCOG scores ≥ 4 (*N* = 109); RCOG scores 2–3 (*N* = 470); RCOG scores ≤ 1 (*N* = 163); H: highrisk;M:medium risk;L:low risk

Between January 2020 and March 2022, our prospective observational study encompassed 742 women who underwent ultrasound examination and D-dimer testing on the first day following cesarean section (CS). Consequently, the study comprised 15 cases of venous thromboembolism (VTE group) and 727 women without VTE (control group). According to the 2015 RCOG scoring, 109 patients scored ≥ 4 points for postpartum VTE risk (high risk), 470 patients scored 2 or 3 points (medium risk), and 163 patients scored 1 point (low risk) (Fig. 1). The mean age of the included patients was 30.71 ± 4.44 years, and the mean D-dimer level on the day after Cesarean section was 4.95 ± 6.64 mg/l. A significant difference in patient age was observed between the VTE group (34.07 ± 3.71 years) and the control group (30.67 ± 4.45 years), *P* < 0.05. Additionally, a significant difference in D-dimer levels was noted between the VTE group (12.08 ± 10.45 mg/l) and the control group (6.05 ± 6.03 mg/l), *P* < 0.05 (Table 1). No maternal deaths occurred in the study cohort. 80% (12/15) of patients diagnosed with VTE fell into the medium-risk category. Detailed information on the 15 postpartum women with thrombosis after CS is presented in Table 2. The overall rate of VTE following cesarean section was 2.02% (15/742), with 66.7% (10/15) of cases being asymptomatic. Seventy-three point 3% (11/15) of VTE diagnoses occurred via ultrasound or CTPA on the first postpartum day. Among the diagnosed cases, five patients presented with pulmonary embolism (PE) combined with deep vein thrombosis (DVT), with 60% (3/5) being asymptomatic; the others exhibited symptoms such as chest tightness, dizziness, lower limb pain, and decreased transcutaneous oxygen saturation. Seven patients were diagnosed with DVT, and only one presented with symptoms of lower limb pain. Finally, one patient diagnosed with ovarian venous thrombosis reported abdominal pain.

**Table 1:** Summarizes the clinical characteristics of the study population (*N* = 742)

	VTE group (*n* = 15)	Control group (*n* = 727)	P
Age (years) ^a^	34.07 ± 3.845	30.67 ± 4.45	0.003
BMI ^a^	27.20 ± 2.85	27.899 ± 3.63	0.459
Gravida ^a^	3.73 ± 1.67	2.73 ± 1.63	0.018
Para ^a^	2.13 ± 0.64	1.74 ± 0.73	0.04
Gestational age ^a^	37.53 ± 1.64	37.87 ± 2.05	0.527
Birth Weight ^a^	3267.69 ± 491.40	3222.69 ± 538.05	0.765
Emergency CS	7(47%)	334(46%)	0.947
Diabetes mellitus	2(13%)	130(18%)	0.631
Hypertensive disorders during pregnancy	1(7%)	80(11%)	0.566
RCOG score			0.09
≥ 4	3 (20%)	106 (14.6%)	
2 and 3	12 (80%)	458 (63%)	
1	0 (0)	163 (22.4%)	
Results of CUS (Day 1)			0.00
VTE	10 (66.7%)	0	
Abnormal	2 (13.3%)	61 (8.4%)	
Normal	3 (10%)	666 (91.6%)	
D-dimer ≥ 3 (Day 1) ^b^			0.001
Yes	14 (93.3%)	478 (65.7%)	
No	1 (6.7%)	249 (34.3%)	
D-dimer (mg/l EFU) (Day 1) ^a^	12.08 ± 10.45	6.05 ± 6.03	0.049

^a^ Student’s t tests, ^b^ Fisher’s exact tests, A chi-squared test was used to analysis categorical variables

**Table 2:** Summarizes the clinical characteristics of VTE cases (*n* = 15)

	level	Overall
Symptoms	no	10(66.7%)
	yes	5(33.3%)
Time of diagnosis	≤ 1 day	11(73.3%)
	>1 day	4(26.7%)
Results of CUS	VTE	10(66.7%)
	hypercoagulative	2(13.3%)
	blood vessels pristine	3(20.0%)
D-dimer	≥ 3	14(93.3%)
	<3	1(6.7%)
2015 RCOG score	2–3	12(80.0%)
	≥ 4	3(20.0%)

Among the 742 postpartum women included in our study, 492 parturients exhibited a postpartum D-dimer ≥ 3 mg/l, with 12.8% (14/492) of them eventually diagnosed with venous thromboembolism (VTE). A total of 671 parturients displayed normal ultrasound findings on the first day following cesarean section (CS), yet 0.45% (3/671) of them were ultimately diagnosed with VTE. In contrast, 41 parturients, despite receiving pharmacological VTE prophylaxis with LMWH, exhibited abnormal ultrasound findings and a D-dimer ≥ 3 mg/l on the first day after CS, resulting in a 4.9% (2/41) VTE diagnosis within the high-risk group. Furthermore, 30.86% of parturients (229/742) demonstrated normal ultrasound results and D-dimer levels < 3 mg/l on the first day after CS, with no VTE occurrences during postpartum follow-up. The sensitivity, specificity, positive predictive value, and negative predictive value of abnormal CUS were 80%, 91.6%, 16.4%, and 99.6%, respectively. For D-dimer ≥ 3 mg/l, the corresponding values were 93.3%, 34.3%, 2.8%, and 99.6%, while for abnormal CUS accompanied by D-dimer ≥ 3 mg/l, they were 73.3%, 94.4%, 21.2%, and 99.4%.

In accordance with RCOG’s recommendation, 78.03% (579/742) of women undergoing cesarean delivery should receive prophylactic anticoagulation. However, as per our department’s VTE prevention program, only 20.62% of patients (153/742) received anticoagulation following cesarean section.

## Discussion

We observed a venous thromboembolism (VTE) rate of 2.02% (15/742) in cesarean section (CS) cases, with 66.7% (10/15) being asymptomatic. Among those diagnosed with VTE, 80% (12/15) fell into the medium-risk category, and 73.3% (11/15) received their diagnosis on the first postpartum day, with 90.91% (10/11) of these cases being detected through CUS. Employing our strategy, all VTE cases were diagnosed during hospitalization, and no new occurrences were identified during the follow-up period extending up to 42 days postpartum. Only 20.62% (153/742) of parturients met our criteria for prophylactic anticoagulation, whereas 78.03% (579/742) aligned with the recommendations of the Royal College of Obstetricians and Gynaecologists (RCOG).

We systematically conduct thrombosis screening using CUS and D-dimer monitoring after CS delivery, revealing a symptomatic VTE incidence of 0.67% (5/742). This rate is notably higher than reported in previous studies [19–21], where the incidence of symptomatic VTE was 0.18%. The discrepancy can be attributed to our focus solely on CS puerperium, representing the demographic with the highest VTE susceptibility during the peak risk period. Our strategy, emphasizing early detection and treatment of asymptomatic thrombosis, proves effective in preventing severe thrombotic disease.

Prompt and accurate diagnosis of venous thromboembolism (VTE) in the puerperium is crucial, as a missed or delayed diagnosis could pose a threat to maternal lives. The Royal College of Obstetricians and Gynaecologists (RCOG) risk assessment model stands out as one of the more intricate scales for pregnancy-related VTE. Its effectiveness in reducing maternal mortality from pulmonary embolism (PE) in the United Kingdom has been demonstrated. Traditionally, there is a perception that Asian populations exhibit a relatively lower risk of VTE compared to individuals in Western countries [21]. According to RCOG guidelines, thromboprophylaxis for at least 10 days is recommended if the total score is ≥ 2 postnatally [22]. Consequently, 78.03% of women after cesarean delivery are recommended prophylactic anticoagulants. While postpartum thrombus prevention has garnered attention among obstetricians in China, the widespread adoption of this prevention strategy remains limited. Despite the RCOG protocol’s ability to identify the majority of patients eventually developing postpartum VTE, many obstetricians in Asian countries are concerned about potential overuse of anticoagulant drugs in women less likely to develop postpartum VTE. In our study, anticoagulation was administered to only 20.62% (153/742) of patients after cesarean section. We observed that intermediate-risk postpartum women accounted for 63.3% (470/742) of CS deliveries without VTE, meeting the criteria for thromboprophylaxis according to RCOG recommendations. In our strategy, only 4.3% (32/742) of middle-risk and low-risk women without VTE received prophylactic anticoagulants, and nine VTE cases were treated with therapeutic anticoagulants. Subsequent monitoring with D-dimer revealed only three cases (3/470) of VTE in those without prophylactic anticoagulants (one asymptomatic PE, one ovarian venous thrombosis, and one lower extremity intermuscular vein thrombosis). In the high-risk group, despite the use of prophylactic anticoagulants in all patients, one case was diagnosed with VTE on the first day after CS, and two cases were diagnosed in the following days. These latter two cases were identified among those with abnormal ultrasound findings and D-dimer levels ≥ 3 mg/l on the first day after CS. For the high-risk group, therapeutic anticoagulants may be more appropriate for those with abnormal ultrasound findings and D-dimer levels ≥ 3 mg/l. Risk-stratified LMWH-based thromboprophylaxis in a general obstetric population has been associated with increased bleeding complications, particularly after cesarean delivery [14]. Therefore, higher-quality evidence is needed to guide best practices for preventing obstetric VTE, with the ideal thromboprophylaxis strategy having an acceptable risk/benefit ratio. There is limited evidence describing the effectiveness of LMWH-based thromboprophylaxis in parturients undergoing cesarean section in the Chinese Han population.

D-dimer serves as a crucial indicator in the differential diagnosis of venous thromboembolism (VTE). Nevertheless, its utility for guiding diagnostic decisions in pregnancy is constrained by physiological increases during pregnancy, peaking in the early puerperium [23], and exhibiting high intra-individual biological variation [22]. Consequently, the value of D-dimer in pregnancy remains a subject of controversy. No guidelines recommend relying solely on D-dimer as a reference indicator for screening, diagnosing, preventing, or treating VTE in pregnant women. Notably, we observed a significant difference in D-dimer levels between the VTE group and the control group. In cases where patients exhibit normal compression CUS but abnormal D-dimer levels, dynamic monitoring of D-dimer is advisable, and a review of CUS should be considered if a rebound is observed.

Our thrombus prevention strategy post-cesarean section (CS) facilitated the early diagnosis and treatment of asymptomatic thrombi, preventing the occurrence of potentially fatal thrombi. Although this approach may lead to increased detection of micro-deep vein thromboses (DVTs) and necessitate a shift in LMWH dosage from prophylactic to therapeutic, the benefit lies in averting the development of VTE, making it advantageous for patients.

Under our strategy, 100% of deep venous thrombosis cases were diagnosed. This approach has demonstrated safety, with 26.95% (200) of parturients exhibiting normal lower limb ultrasound and D-dimer < 3, along with an RCOG score < 4 at the initial postpartum assessment. This subgroup, left untreated with thromboprophylaxis, experienced no occurrences of venous thromboembolism (VTE) during the postpartum follow-up.

Our approach to VTE management following cesarean section (CS) appears to be safe, effective, and cost-efficient within the Chinese Han population. However, it is imperative to note that this was a single-center observational study with a limited duration and a small sample size. To validate these findings, multi-center, large-sample studies are warranted.

## Conclusion

In summary, our study affirms that, following CS delivery, the combination of D-dimer monitoring and timely routine CUS of lower limbs facilitates early diagnosis and treatment of VTE. Moreover, this approach significantly reduces the use of LMWH in the Chinese Han population.

## Data Availability

All data generated or analyzed during this study are included in this article.

## Abbreviations

VTE: venous thromboembolism
CS: cesarean section
DVT: deep vein thrombosis
PE: pulmonary embolism
LMWH: low-molecular-weight heparin
CUS: compression ultrasonography
CTPA: computed-tomography pulmonary angiography
EFU: fibrinogen-equivalent units
BMI: Body Mass Index

## References

[1] Bitsadze V, Khizroeva J, Alexander M, Elalamy I (2022). Venous thrombosis risk factors in pregnant women. J Perinat Med.

[2] Park JE, Park Y, Yuk JS (2021). Incidence of and risk factors for thromboembolism during pregnancy and postpartum: a 10-year nationwide population-based study. Taiwan J Obstet Gynecol.

[3] Blanco-Molina A, Trujillo-Santos J, Criado J, Lopez L, Lecumberri R, Gutierrez R (2007). Venous thromboembolism during pregnancy or postpartum: findings from the RIETE Registry. Thromb Haemost.

[4] Zhou Q, Zhao Z, Xu J, Xiong Y, Li X (2022). Hospital Variation and Associated Organizational factors of pregnancy-related venous thromboembolism in China. Clin Appl Thromb Hemost.

[5] Cantwell R, Clutton-Brock T, Cooper G, Dawson A, Drife J, Garrod D (2011). Saving mothers’ lives: reviewing maternal deaths to make motherhood safer: 2006–2008. The Eighth Report of the confidential enquiries into maternal deaths in the United Kingdom. BJOG.

[6] Krenitsky N, Friedman AM, Yu K, Gyamfi-Bannerman C, Williams-Kane J, O’Shaugnessy F (2022). Trends in venous thromboembolism and Associated Risk factors during delivery hospitalizations from 2000 to 2018. Obstet Gynecol.

[7] Seeho S, Nassar N (2016). Thromboprophylaxis after caesarean: when even the ‘experts’ disagree. BJOG.

[8] Friedman AM (2021). Obstetric venous thromboembolism prophylaxis, risk factors and outcomes. Curr Opin Obstet Gynecol.

[9] Goodacre S, Hunt B, Nelson-Piercy C (2019). Diagnosis of pulmonary embolism during pregnancy. Ann Intern Med.

[10] Heit JA, Kobbervig CE, James AH, Petterson TM, Bailey KR, Melton LJ (2005). 3rd. Trends in the incidence of venous thromboembolism during pregnancy or postpartum: a 30-year population-based study. Ann Intern Med.

[11] Jackson E, Curtis KM, Gaffield ME (2011). Risk of venous thromboembolism during the postpartum period: a systematic review. Obstet Gynecol.

[12] Blondon M, Casini A, Hoppe KK, Boehlen F, Righini M, Smith NL (2016). Risks of venous thromboembolism after cesarean sections: a Meta-analysis. Chest.

[13] Alsheef MA, Alabbad AM, Albassam RA, Alarfaj RM, Zaidi ARZ, Al-Arfaj O (2020). Pregnancy and venous thromboembolism: risk factors, trends, Management, and Mortality. Biomed Res Int.

[14] Ge YZ, Zhang C, Cai YQ, Huang HF (2021). Application of the RCOG Risk Assessment Model for evaluating Postpartum venous thromboembolism in Chinese women: a case-control study. Med Sci Monit.

[15] Kotaska A (2018). Postpartum venous thromboembolism prophylaxis may cause more harm than benefit: a critical analysis of international guidelines through an evidence-based lens. BJOG.

[16] Friedman AM, D’Alton ME (2021). Expert review: prevention of obstetrical venous thromboembolism. Am J Obstet Gynecol.

[17] Ernst DM, Oporto JI, Zuñiga PA, Pereira JI, Vera CM, Carvajal JA (2021). Maternal and perinatal outcomes of a venous thromboembolism high-risk cohort using a multidisciplinary treatment approach. Int J Gynaecol Obstet.

[18] Kalaitzopoulos DR, Panagopoulos A, Samant S, Ghalib N, Kadillari J, Daniilidis A (2022). Management of venous thromboembolism in pregnancy. Thromb Res.

[19] Pon TK, Wessel N, Cagonot V, Delmonte R, Roach D, Finta L (2019). Utilization of venous thromboembolism prophylaxis in American hospitalized pregnant women undergoing cesarean section. Int J Clin Pharm.

[20] Zhao Z, Zhou Q, Li X (2021). Missed opportunities for venous thromboembolism prophylaxis during pregnancy and the postpartum period: evidence from mainland China in 2019. BMC Pregnancy Childbirth.

[21] Hong J, Lee JH, Yhim HY, Choi WI, Bang SM, Lee H (2018). Incidence of venous thromboembolism in Korea from 2009 to 2013. PLoS ONE.

[22] Hedengran KK, Andersen MR, Stender S, Szecsi PB (2016). Large D-Dimer fluctuation in normal pregnancy: a longitudinal cohort study of 4,117 samples from 714 healthy Danish women. Obstet Gynecol Int.

[23] Bitsadze V, Khizroeva J, Elalamy I, Alexander M. Venous thrombosis risk factors in pregnant women. J Perinat Med. 2020. 10.1515/jpm-2020-0011 33098632

